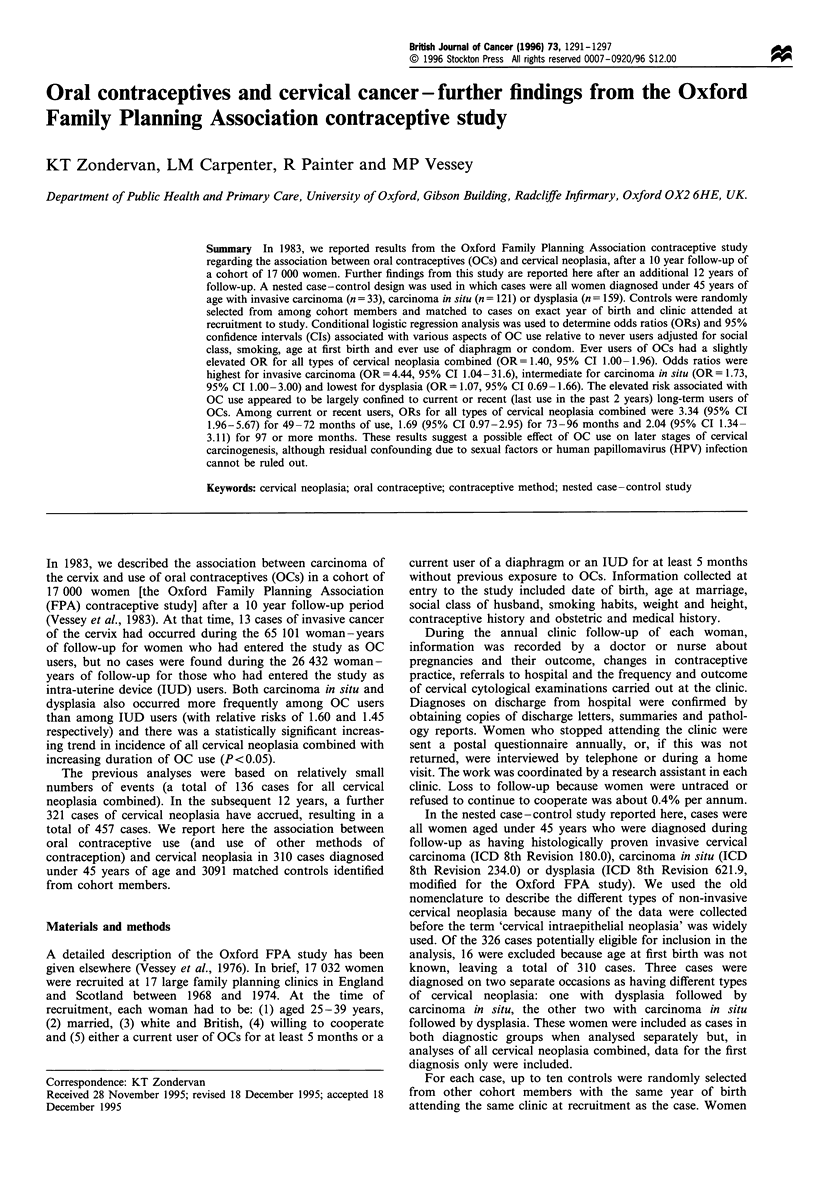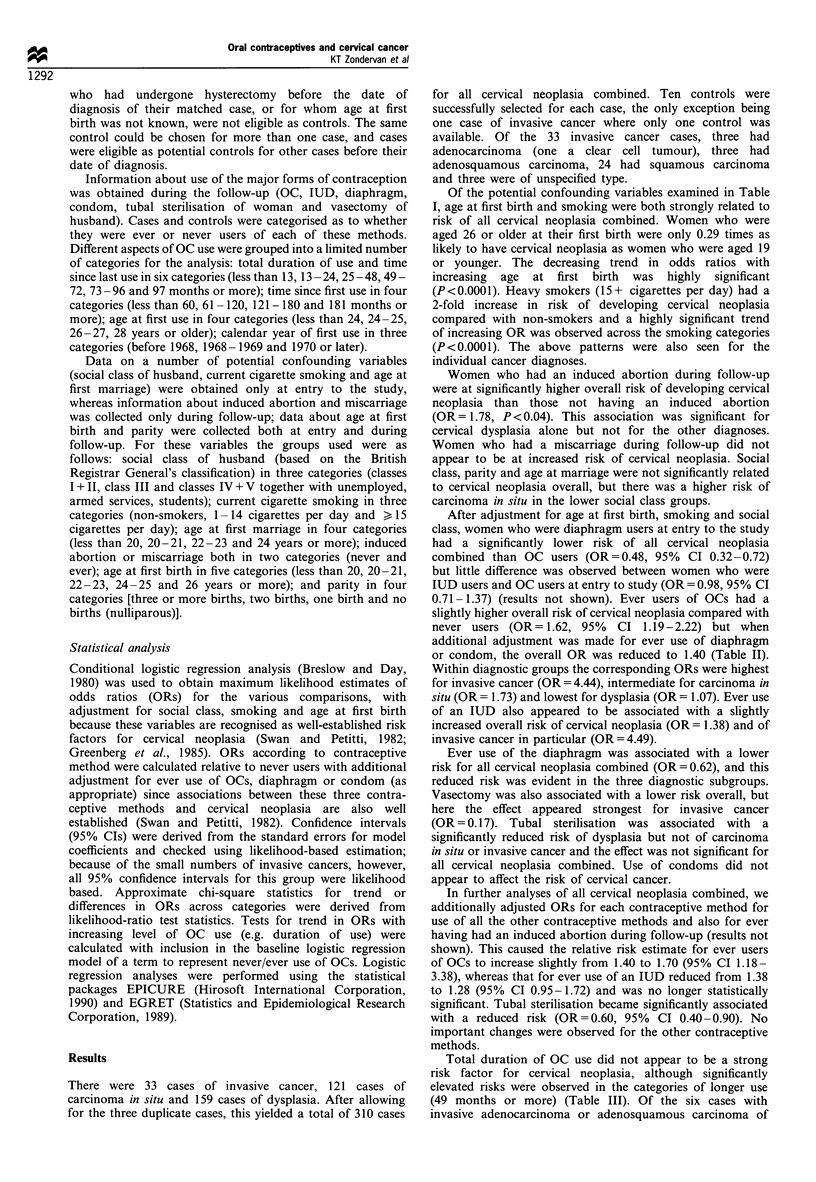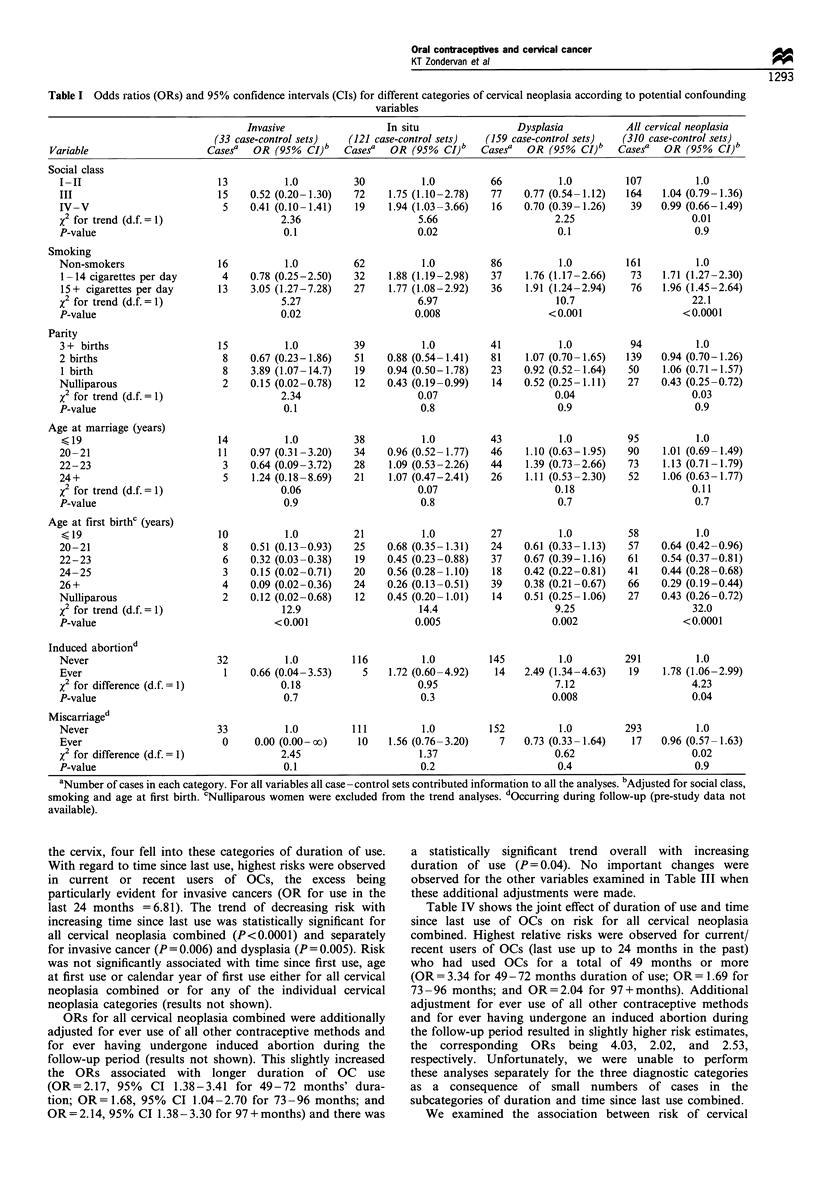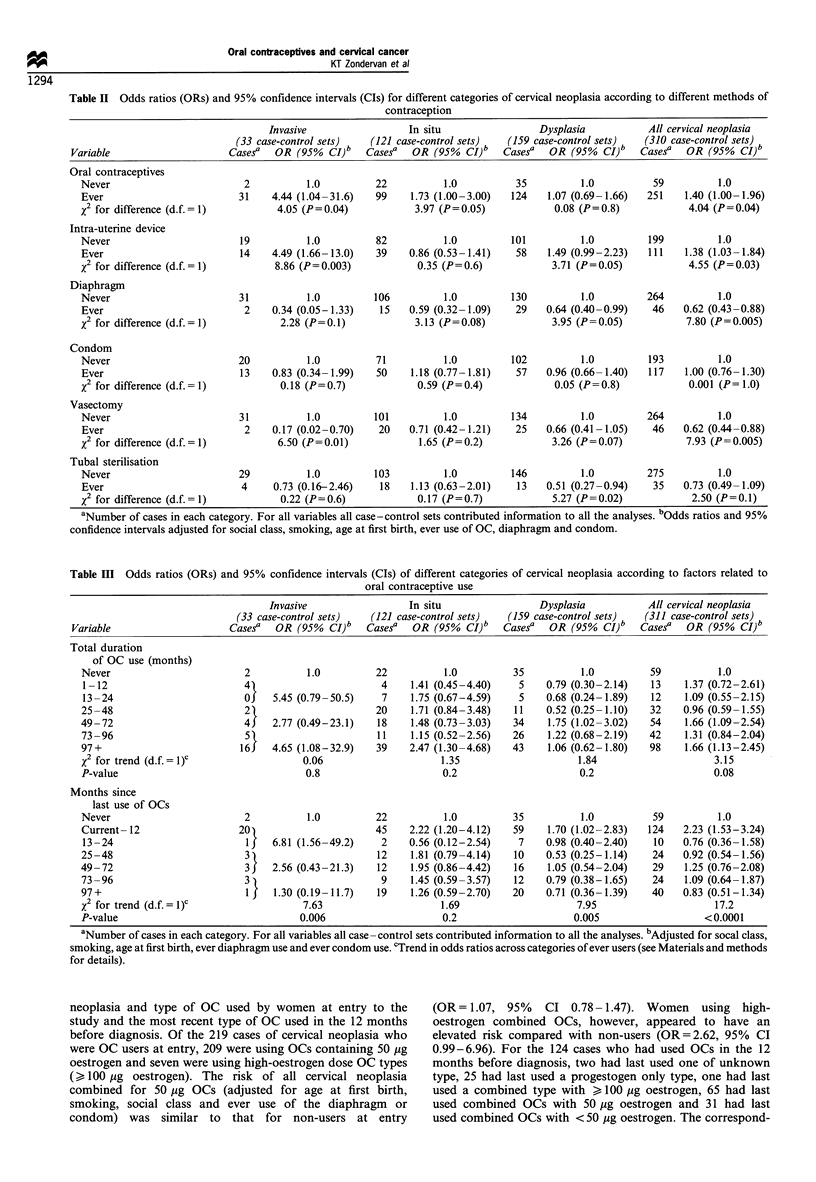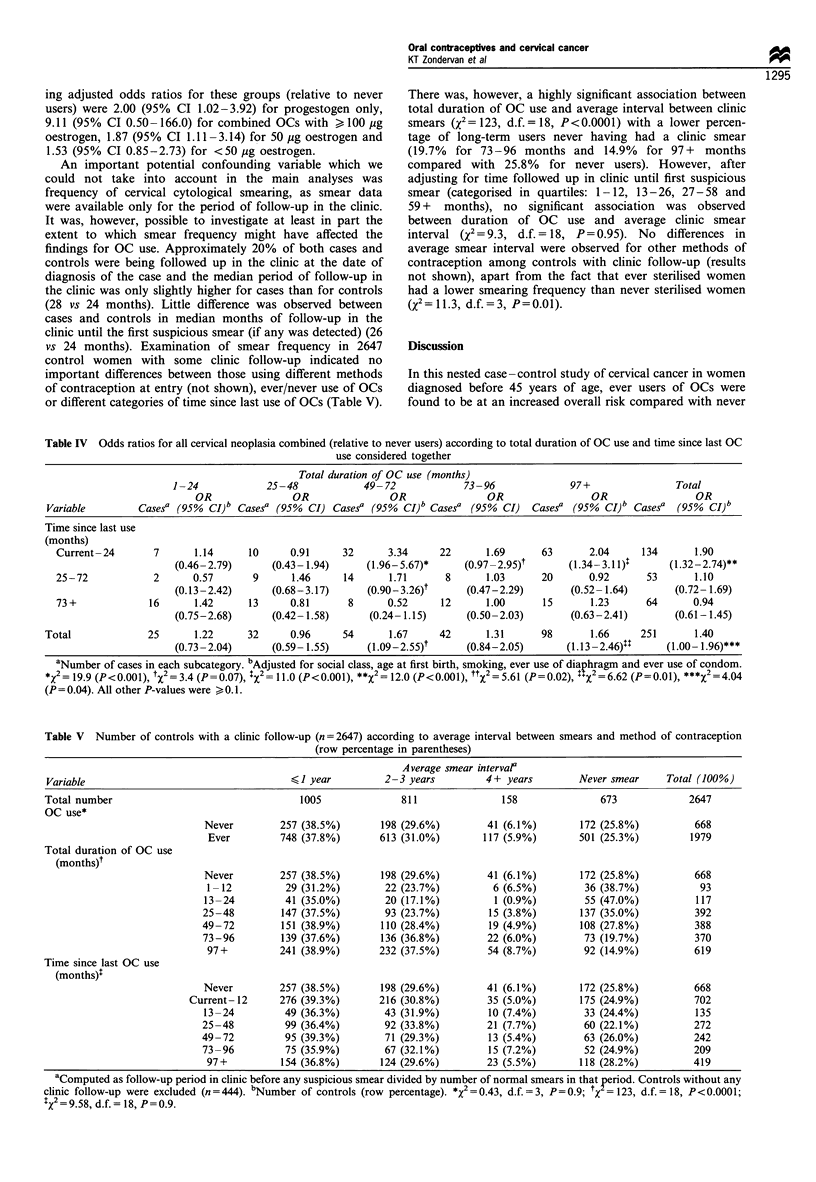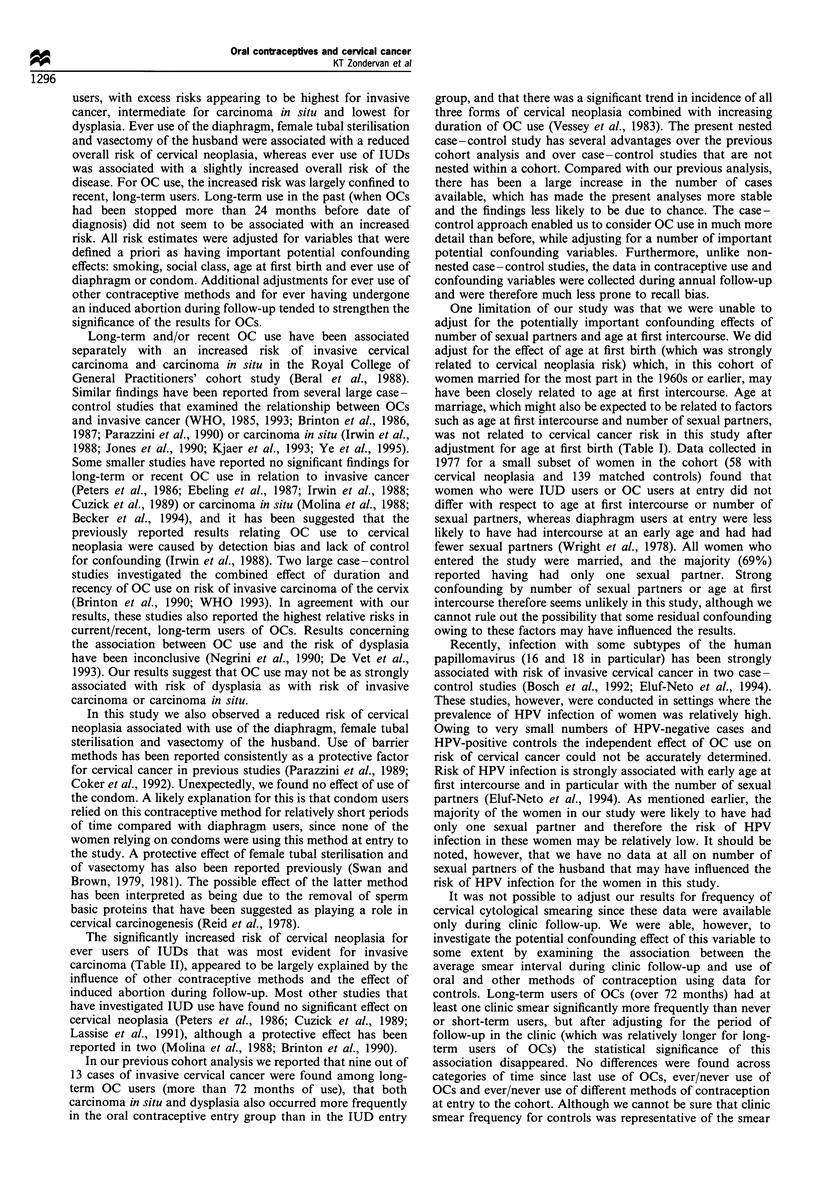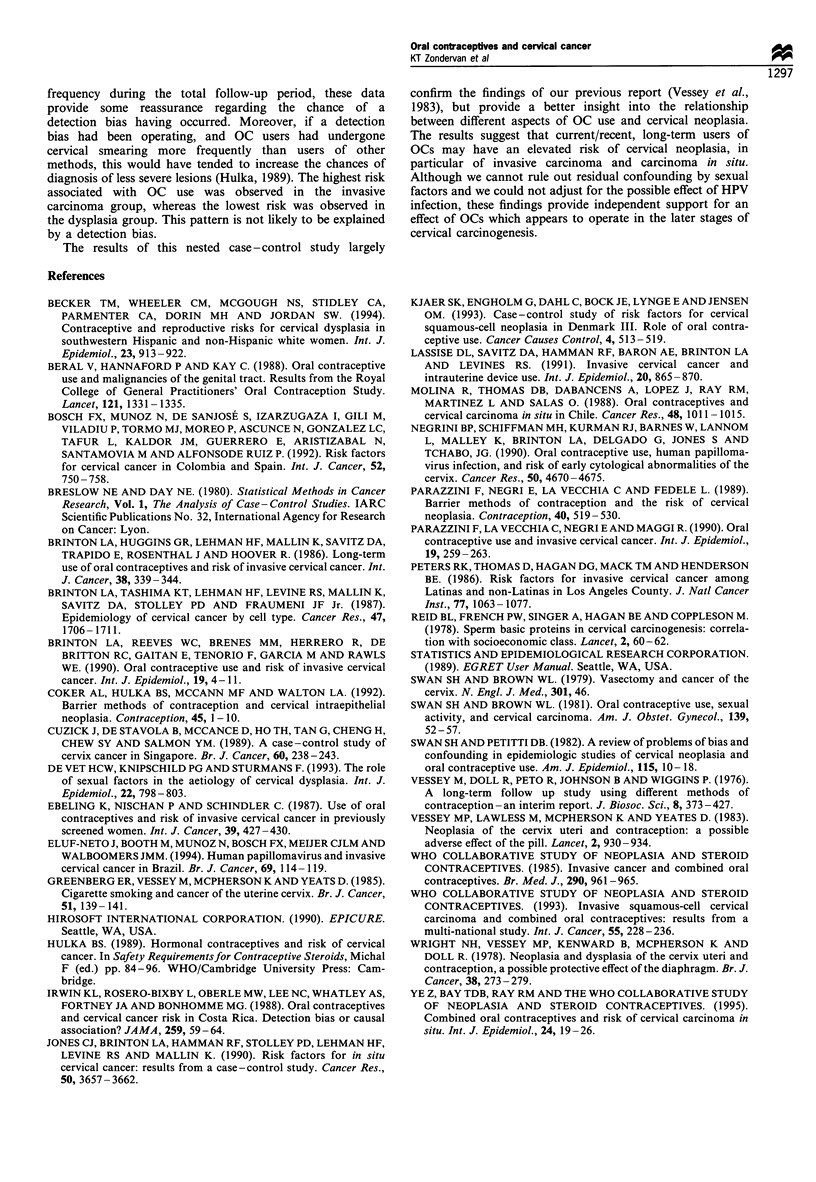# Oral contraceptives and cervical cancer--further findings from the Oxford Family Planning Association contraceptive study.

**DOI:** 10.1038/bjc.1996.247

**Published:** 1996-05

**Authors:** K. T. Zondervan, L. M. Carpenter, R. Painter, M. P. Vessey

**Affiliations:** Department of Public Health and Primary Care, University of Oxford, Radcliffe Infirmary, UK.

## Abstract

In 1983, we reported results from the Oxford Family Planning Association contraceptive study regarding the association between oral contraceptives (OCs) and cervical neoplasia, after a 10 year follow-up of a cohort of 17,000 women. Further findings from this study are reported here after an additional 12 years of follow-up. A nested case--control design was used in which cases were all women diagnosed under 45 years of age with invasive carcinoma (n = 33), carcinoma in situ (n = 121) or dysplasia (n = 159). Controls were randomly selected from among cohort members and matched to cases on exact year of birth and clinic attended at recruitment to study. Conditional logistic regression analysis was used to determine odds ratios (ORs) and 95% confidence intervals (CIs) associated with various aspects of OC use relative to never users adjusted for social class, smoking, age at first birth and ever use of diaphragm or condom. Ever users of OCs had a slightly elevated OR for all types of cervical neoplasia combined (OR = 1.40, 95% CI 1.00-1.96). Odds ratios were highest for invasive carcinoma (OR = 4.44, 95% CI 1.04-31.6), intermediate for carcinoma in situ (OR = 1.73, 95% CI 1.00-3.00) and lowest for dysplasia (OR = 1.07, 95% CI 0.69-1.66). The elevated risk associated with OC use appeared to be largely confined to current or recent (last use in the past 2 years) long-term users of OCs. Among current or recent users, ORs for all types of cervical neoplasia combined were 3.34 (95% CI 1.96-5.67) for 49-72 months of use, 1.69 (95% CI 0.97-2.95) for 73-96 months and 2.04 (95% CI 1.34-3.11) for 97 or more months. These results suggest a possible effect of OC use on later stages of cervical carcinogenesis, although residual confounding due to sexual factors or human papillomavirus (HPV) infection cannot be ruled out.


British Journal of Cancer (1996) 73, 1291-1297
© 1996 Stockton Press All rights reserved 0007-0920/96 $12.00

### Oral contraceptives and cervical cancer – further findings from the Oxford Family Planning Association contraceptive study

KT Zondervan, LM Carpenter, R Painter and MP Vessey

*Department of Public Health and Primary Care, University of Oxford, Gibson Building, Radcliffe Infirmary, Oxford OX2 6HE, UK.*

**Summary** In 1983, we reported results from the Oxford Family Planning Association contraceptive study regarding the association between oral contraceptives (OCs) and cervical neoplasia, after a 10 year follow-up of a cohort of 17 000 women. Further findings from this study are reported here after an additional 12 years of follow-up. A nested case–control design was used in which cases were all women diagnosed under 45 years of age with invasive carcinoma ($n$ = 33), carcinoma *in situ* ($n$ = 121) or dysplasia ($n$ = 159). Controls were randomly selected from among cohort members and matched to cases on exact year of birth and clinic attended at recruitment to study. Conditional logistic regression analysis was used to determine odds ratios (ORs) and 95% confidence intervals (CIs) associated with various aspects of OC use relative to never users adjusted for social class, smoking, age at first birth and ever use of diaphragm or condom. Ever users of OCs had a slightly elevated OR for all types of cervical neoplasia combined (OR = 1.40, 95% CI 1.00–1.96). Odds ratios were highest for invasive carcinoma (OR = 4.44, 95% CI 1.04–31.6), intermediate for carcinoma *in situ* (OR = 1.73, 95% CI 1.00–3.00) and lowest for dysplasia (OR = 1.07, 95% CI 0.69–1.66). The elevated risk associated with OC use appeared to be largely confined to current or recent (last use in the past 2 years) long-term users of OCs. Among current or recent users, ORs for all types of cervical neoplasia combined were 3.34 (95% CI 1.96–5.67) for 49–72 months of use, 1.69 (95% CI 0.97–2.95) for 73–96 months and 2.04 (95% CI 1.34–3.11) for 97 or more months. These results suggest a possible effect of OC use on later stages of cervical carcinogenesis, although residual confounding due to sexual factors or human papillomavirus (HPV) infection cannot be ruled out.

**Keywords:** cervical neoplasia; oral contraceptive; contraceptive method; nested case–control study

In 1983, we described the association between carcinoma of the cervix and use of oral contraceptives (OCs) in a cohort of 17 000 women [the Oxford Family Planning Association (FPA) contraceptive study] after a 10 year follow-up period (Vessey *et al*., 1983). At that time, 13 cases of invasive cancer of the cervix had occurred during the 65 101 woman–years of follow-up for women who had entered the study as OC users, but no cases were found during the 26 432 woman–years of follow-up for those who had entered the study as intra-uterine device (IUD) users. Both carcinoma *in situ* and dysplasia also occurred more frequently among OC users than among IUD users (with relative risks of 1.60 and 1.45 respectively) and there was a statistically significant increasing trend in incidence of all cervical neoplasia combined with increasing duration of OC use ($P < 0.05$).

The previous analyses were based on relatively small numbers of events (a total of 136 cases for all cervical neoplasia combined). In the subsequent 12 years, a further 321 cases of cervical neoplasia have accrued, resulting in a total of 457 cases. We report here the association between oral contraceptive use (and use of other methods of contraception) and cervical neoplasia in 310 cases diagnosed under 45 years of age and 3091 matched controls identified from cohort members.

### Materials and methods

A detailed description of the Oxford FPA study has been given elsewhere (Vessey *et al*., 1976). In brief, 17 032 women were recruited at 17 large family planning clinics in England and Scotland between 1968 and 1974. At the time of recruitment, each woman had to be: (1) aged 25–39 years, (2) married, (3) white and British, (4) willing to cooperate and (5) either a current user of OCs for at least 5 months or a current user of a diaphragm or an IUD for at least 5 months without previous exposure to OCs. Information collected at entry to the study included date of birth, age at marriage, social class of husband, smoking habits, weight and height, contraceptive history and obstetric and medical history.

During the annual clinic follow-up of each woman, information was recorded by a doctor or nurse about pregnancies and their outcome, changes in contraceptive practice, referrals to hospital and the frequency and outcome of cervical cytological examinations carried out at the clinic. Diagnoses on discharge from hospital were confirmed by obtaining copies of discharge letters, summaries and pathology reports. Women who stopped attending the clinic were sent a postal questionnaire annually, or, if this was not returned, were interviewed by telephone or during a home visit. The work was coordinated by a research assistant in each clinic. Loss to follow-up because women were untraced or refused to continue to cooperate was about 0.4% per annum.

In the nested case–control study reported here, cases were all women aged under 45 years who were diagnosed during follow-up as having histologically proven invasive cervical carcinoma (ICD 8th Revision 180.0), carcinoma *in situ* (ICD 8th Revision 234.0) or dysplasia (ICD 8th Revision 621.9, modified for the Oxford FPA study). We used the old nomenclature to describe the different types of non-invasive cervical neoplasia because many of the data were collected before the term 'cervical intraepithelial neoplasia' was widely used. Of the 326 cases potentially eligible for inclusion in the analysis, 16 were excluded because age at first birth was not known, leaving a total of 310 cases. Three cases were diagnosed on two separate occasions as having different types of cervical neoplasia: one with dysplasia followed by carcinoma *in situ*, the other two with carcinoma *in situ* followed by dysplasia. These women were included as cases in both diagnostic groups when analysed separately but, in analyses of all cervical neoplasia combined, data for the first diagnosis only were included.

For each case, up to ten controls were randomly selected from other cohort members with the same year of birth attending the same clinic at recruitment as the case. Women

Correspondence: KT Zondervan
Received 28 November 1995; revised 18 December 1995; accepted 18 December 1995

who had undergone hysterectomy before the date of diagnosis of their matched case, or for whom age at first birth was not known, were not eligible as controls. The same control could be chosen for more than one case, and cases were eligible as potential controls for other cases before their date of diagnosis.

Information about use of the major forms of contraception was obtained during the follow-up (OC, IUD, diaphragm, condom, tubal sterilisation of woman and vasectomy of husband). Cases and controls were categorised as to whether they were ever or never users of each of these methods. Different aspects of OC use were grouped into a limited number of categories for the analysis: total duration of use and time since last use in six categories (less than 13, 13–24, 25–48, 49–72, 73–96 and 97 months or more); time since first use in four categories (less than 60, 61–120, 121–180 and 181 months or more); age at first use in four categories (less than 24, 24–25, 26–27, 28 years or older); calendar year of first use in three categories (before 1968, 1968–1969 and 1970 or later).

Data on a number of potential confounding variables (social class of husband, current cigarette smoking and age at first marriage) were obtained only at entry to the study, whereas information about induced abortion and miscarriage was collected only during follow-up; data about age at first birth and parity were collected both at entry and during follow-up. For these variables the groups used were as follows: social class of husband (based on the British Registrar General's classification) in three categories (classes I+II, class III and classes IV+V together with unemployed, armed services, students); current cigarette smoking in three categories (non-smokers, 1–14 cigarettes per day and ⩾15 cigarettes per day); age at first marriage in four categories (less than 20, 20–21, 22–23 and 24 years or more); induced abortion or miscarriage both in two categories (never and ever); age at first birth in five categories (less than 20, 20–21, 22–23, 24–25 and 26 years or more); and parity in four categories [three or more births, two births, one birth and no births (nulliparous)].

### *Statistical analysis*

Conditional logistic regression analysis (Breslow and Day, 1980) was used to obtain maximum likelihood estimates of odds ratios (ORs) for the various comparisons, with adjustment for social class, smoking and age at first birth because these variables are recognised as well-established risk factors for cervical neoplasia (Swan and Petitti, 1982; Greenberg *et al.*, 1985). ORs according to contraceptive method were calculated relative to never users with additional adjustment for ever use of OCs, diaphragm or condom (as appropriate) since associations between these three contraceptive methods and cervical neoplasia are also well established (Swan and Petitti, 1982). Confidence intervals (95% CIs) were derived from the standard errors for model coefficients and checked using likelihood-based estimation; because of the small numbers of invasive cancers, however, all 95% confidence intervals for this group were likelihood based. Approximate chi-square statistics for trend or differences in ORs across categories were derived from likelihood-ratio test statistics. Tests for trend in ORs with increasing level of OC use (e.g. duration of use) were calculated with inclusion in the baseline logistic regression model of a term to represent never/ever use of OCs. Logistic regression analyses were performed using the statistical packages EPICURE (Hirosoft International Corporation, 1990) and EGRET (Statistics and Epidemiological Research Corporation, 1989).

### Results

There were 33 cases of invasive cancer, 121 cases of carcinoma *in situ* and 159 cases of dysplasia. After allowing for the three duplicate cases, this yielded a total of 310 cases for all cervical neoplasia combined. Ten controls were successfully selected for each case, the only exception being one case of invasive cancer where only one control was available. Of the 33 invasive cancer cases, three had adenocarcinoma (one a clear cell tumour), three had adenosquamous carcinoma, 24 had squamous carcinoma and three were of unspecified type.

Of the potential confounding variables examined in Table I, age at first birth and smoking were both strongly related to risk of all cervical neoplasia combined. Women who were aged 26 or older at their first birth were only 0.29 times as likely to have cervical neoplasia as women who were aged 19 or younger. The decreasing trend in odds ratios with increasing age at first birth was highly significant ($P<0.0001$). Heavy smokers (15+ cigarettes per day) had a 2-fold increase in risk of developing cervical neoplasia compared with non-smokers and a highly significant trend of increasing OR was observed across the smoking categories ($P<0.0001$). The above patterns were also seen for the individual cancer diagnoses.

Women who had an induced abortion during follow-up were at significantly higher overall risk of developing cervical neoplasia than those not having an induced abortion (OR = 1.78, $P<0.04$). This association was significant for cervical dysplasia alone but not for the other diagnoses. Women who had a miscarriage during follow-up did not appear to be at increased risk of cervical neoplasia. Social class, parity and age at marriage were not significantly related to cervical neoplasia overall, but there was a higher risk of carcinoma *in situ* in the lower social class groups.

After adjustment for age at first birth, smoking and social class, women who were diaphragm users at entry to the study had a significantly lower risk of all cervical neoplasia combined than OC users (OR = 0.48, 95% CI 0.32–0.72) but little difference was observed between women who were IUD users and OC users at entry to study (OR = 0.98, 95% CI 0.71–1.37) (results not shown). Ever users of OCs had a slightly higher overall risk of cervical neoplasia compared with never users (OR = 1.62, 95% CI 1.19–2.22) but when additional adjustment was made for ever use of diaphragm or condom, the overall OR was reduced to 1.40 (Table II). Within diagnostic groups the corresponding ORs were highest for invasive cancer (OR = 4.44), intermediate for carcinoma *in situ* (OR = 1.73) and lowest for dysplasia (OR = 1.07). Ever use of an IUD also appeared to be associated with a slightly increased overall risk of cervical neoplasia (OR = 1.38) and of invasive cancer in particular (OR = 4.49).

Ever use of the diaphragm was associated with a lower risk for all cervical neoplasia combined (OR = 0.62), and this reduced risk was evident in the three diagnostic subgroups. Vasectomy was also associated with a lower risk overall, but here the effect appeared strongest for invasive cancer (OR = 0.17). Tubal sterilisation was associated with a significantly reduced risk of dysplasia but not of carcinoma *in situ* or invasive cancer and the effect was not significant for all cervical neoplasia combined. Use of condoms did not appear to affect the risk of cervical cancer.

In further analyses of all cervical neoplasia combined, we additionally adjusted ORs for each contraceptive method for use of all the other contraceptive methods and also for ever having had an induced abortion during follow-up (results not shown). This caused the relative risk estimate for ever users of OCs to increase slightly from 1.40 to 1.70 (95% CI 1.18–3.38), whereas that for ever use of an IUD reduced from 1.38 to 1.28 (95% CI 0.95–1.72) and was no longer statistically significant. Tubal sterilisation became significantly associated with a reduced risk (OR = 0.60, 95% CI 0.40–0.90). No important changes were observed for the other contraceptive methods.

Total duration of OC use did not appear to be a strong risk factor for cervical neoplasia, although significantly elevated risks were observed in the categories of longer use (49 months or more) (Table III). Of the six cases with invasive adenocarcinoma or adenosquamous carcinoma of

**Table I** Odds ratios (ORs) and 95% confidence intervals (CIs) for different categories of cervical neoplasia according to potential confounding variables

| *Variable* | *Invasive (33 case-control sets)* Cases[a] | OR (95% CI)[b] | In situ (121 case-control sets) Cases[a] | OR (95% CI)[b] | *Dysplasia (159 case-control sets)* Cases[a] | OR (95% CI)[b] | *All cervical neoplasia (310 case-control sets)* Cases[a] | OR (95% CI)[b] |
|---|---|---|---|---|---|---|---|---|
| Social class | | | | | | | | |
| I–II | 13 | 1.0 | 30 | 1.0 | 66 | 1.0 | 107 | 1.0 |
| III | 15 | 0.52 (0.20–1.30) | 72 | 1.75 (1.10–2.78) | 77 | 0.77 (0.54–1.12) | 164 | 1.04 (0.79–1.36) |
| IV–V | 5 | 0.41 (0.10–1.41) | 19 | 1.94 (1.03–3.66) | 16 | 0.70 (0.39–1.26) | 39 | 0.99 (0.66–1.49) |
| $\chi^2$ for trend (d.f. = 1) | | 2.36 | | 5.66 | | 2.25 | | 0.01 |
| *P*-value | | 0.1 | | 0.02 | | 0.1 | | 0.9 |
| Smoking | | | | | | | | |
| Non-smokers | 16 | 1.0 | 62 | 1.0 | 86 | 1.0 | 161 | 1.0 |
| 1–14 cigarettes per day | 4 | 0.78 (0.25–2.50) | 32 | 1.88 (1.19–2.98) | 37 | 1.76 (1.17–2.66) | 73 | 1.71 (1.27–2.30) |
| 15+ cigarettes per day | 13 | 3.05 (1.27–7.28) | 27 | 1.77 (1.08–2.92) | 36 | 1.91 (1.24–2.94) | 76 | 1.96 (1.45–2.64) |
| $\chi^2$ for trend (d.f. = 1) | | 5.27 | | 6.97 | | 10.7 | | 22.1 |
| *P*-value | | 0.02 | | 0.008 | | <0.001 | | <0.0001 |
| Parity | | | | | | | | |
| 3+ births | 15 | 1.0 | 39 | 1.0 | 41 | 1.0 | 94 | 1.0 |
| 2 births | 8 | 0.67 (0.23–1.86) | 51 | 0.88 (0.54–1.41) | 81 | 1.07 (0.70–1.65) | 139 | 0.94 (0.70–1.26) |
| 1 birth | 8 | 3.89 (1.07–14.7) | 19 | 0.94 (0.50–1.78) | 23 | 0.92 (0.52–1.64) | 50 | 1.06 (0.71–1.57) |
| Nulliparous | 2 | 0.15 (0.02–0.78) | 12 | 0.43 (0.19–0.99) | 14 | 0.52 (0.25–1.11) | 27 | 0.43 (0.25–0.72) |
| $\chi^2$ for trend (d.f. = 1) | | 2.34 | | 0.07 | | 0.04 | | 0.03 |
| *P*-value | | 0.1 | | 0.8 | | 0.9 | | 0.9 |
| Age at marriage (years) | | | | | | | | |
| ⩽19 | 14 | 1.0 | 38 | 1.0 | 43 | 1.0 | 95 | 1.0 |
| 20–21 | 11 | 0.97 (0.31–3.20) | 34 | 0.96 (0.52–1.77) | 46 | 1.10 (0.63–1.95) | 90 | 1.01 (0.69–1.49) |
| 22–23 | 3 | 0.64 (0.09–3.72) | 28 | 1.09 (0.53–2.26) | 44 | 1.39 (0.73–2.66) | 73 | 1.13 (0.71–1.79) |
| 24+ | 5 | 1.24 (0.18–8.69) | 21 | 1.07 (0.47–2.41) | 26 | 1.11 (0.53–2.30) | 52 | 1.06 (0.63–1.77) |
| $\chi^2$ for trend (d.f. = 1) | | 0.06 | | 0.07 | | 0.18 | | 0.11 |
| *P*-value | | 0.9 | | 0.8 | | 0.7 | | 0.7 |
| Age at first birth[c] (years) | | | | | | | | |
| ⩽19 | 10 | 1.0 | 21 | 1.0 | 27 | 1.0 | 58 | 1.0 |
| 20–21 | 8 | 0.51 (0.13–0.93) | 25 | 0.68 (0.35–1.31) | 24 | 0.61 (0.33–1.13) | 57 | 0.64 (0.42–0.96) |
| 22–23 | 6 | 0.32 (0.03–0.38) | 19 | 0.45 (0.23–0.88) | 37 | 0.67 (0.39–1.16) | 61 | 0.54 (0.37–0.81) |
| 24–25 | 3 | 0.15 (0.02–0.71) | 20 | 0.56 (0.28–1.10) | 18 | 0.42 (0.22–0.81) | 41 | 0.44 (0.28–0.68) |
| 26+ | 4 | 0.09 (0.02–0.36) | 24 | 0.26 (0.13–0.51) | 39 | 0.38 (0.21–0.67) | 66 | 0.29 (0.19–0.44) |
| Nulliparous | 2 | 0.12 (0.02–0.68) | 12 | 0.45 (0.20–1.01) | 14 | 0.51 (0.25–1.06) | 27 | 0.43 (0.26–0.72) |
| $\chi^2$ for trend (d.f. = 1) | | 12.9 | | 14.4 | | 9.25 | | 32.0 |
| *P*-value | | <0.001 | | 0.005 | | 0.002 | | <0.0001 |
| Induced abortion[d] | | | | | | | | |
| Never | 32 | 1.0 | 116 | 1.0 | 145 | 1.0 | 291 | 1.0 |
| Ever | 1 | 0.66 (0.04–3.53) | 5 | 1.72 (0.60–4.92) | 14 | 2.49 (1.34–4.63) | 19 | 1.78 (1.06–2.99) |
| $\chi^2$ for difference (d.f. = 1) | | 0.18 | | 0.95 | | 7.12 | | 4.23 |
| *P*-value | | 0.7 | | 0.3 | | 0.008 | | 0.04 |
| Miscarriage[d] | | | | | | | | |
| Never | 33 | 1.0 | 111 | 1.0 | 152 | 1.0 | 293 | 1.0 |
| Ever | 0 | 0.00 (0.00–∞) | 10 | 1.56 (0.76–3.20) | 7 | 0.73 (0.33–1.64) | 17 | 0.96 (0.57–1.63) |
| $\chi^2$ for difference (d.f. = 1) | | 2.45 | | 1.37 | | 0.62 | | 0.02 |
| *P*-value | | 0.1 | | 0.2 | | 0.4 | | 0.9 |

[a]Number of cases in each category. For all variables all case–control sets contributed information to all the analyses. [b]Adjusted for social class, smoking and age at first birth. [c]Nulliparous women were excluded from the trend analyses. [d]Occurring during follow-up (pre-study data not available).

the cervix, four fell into these categories of duration of use. With regard to time since last use, highest risks were observed in current or recent users of OCs, the excess being particularly evident for invasive cancers (OR for use in the last 24 months =6.81). The trend of decreasing risk with increasing time since last use was statistically significant for all cervical neoplasia combined ($P<0.0001$) and separately for invasive cancer ($P=0.006$) and dysplasia ($P=0.005$). Risk was not significantly associated with time since first use, age at first use or calendar year of first use either for all cervical neoplasia combined or for any of the individual cervical neoplasia categories (results not shown).

ORs for all cervical neoplasia combined were additionally adjusted for ever use of all other contraceptive methods and for ever having undergone induced abortion during the follow-up period (results not shown). This slightly increased the ORs associated with longer duration of OC use (OR=2.17, 95% CI 1.38–3.41 for 49–72 months' duration; OR=1.68, 95% CI 1.04–2.70 for 73–96 months; and OR=2.14, 95% CI 1.38–3.30 for 97+months) and there was a statistically significant trend overall with increasing duration of use ($P=0.04$). No important changes were observed for the other variables examined in Table III when these additional adjustments were made.

Table IV shows the joint effect of duration of use and time since last use of OCs on risk for all cervical neoplasia combined. Highest relative risks were observed for current/recent users of OCs (last use up to 24 months in the past) who had used OCs for a total of 49 months or more (OR=3.34 for 49–72 months duration of use; OR=1.69 for 73–96 months; and OR=2.04 for 97+months). Additional adjustment for ever use of all other contraceptive methods and for ever having undergone an induced abortion during the follow-up period resulted in slightly higher risk estimates, the corresponding ORs being 4.03, 2.02, and 2.53, respectively. Unfortunately, we were unable to perform these analyses separately for the three diagnostic categories as a consequence of small numbers of cases in the subcategories of duration and time since last use combined.

We examined the association between risk of cervical

**Table II** Odds ratios (ORs) and 95% confidence intervals (CIs) for different categories of cervical neoplasia according to different methods of contraception

| Variable | Invasive (33 case-control sets) Cases[a] | OR (95% CI)[b] | In situ (121 case-control sets) Cases[a] | OR (95% CI)[b] | Dysplasia (159 case-control sets) Cases[a] | OR (95% CI)[b] | All cervical neoplasia (310 case-control sets) Cases[a] | OR (95% CI)[b] |
|---|---|---|---|---|---|---|---|---|
| Oral contraceptives | | | | | | | | |
| Never | 2 | 1.0 | 22 | 1.0 | 35 | 1.0 | 59 | 1.0 |
| Ever | 31 | 4.44 (1.04–31.6) | 99 | 1.73 (1.00–3.00) | 124 | 1.07 (0.69–1.66) | 251 | 1.40 (1.00–1.96) |
| $\chi^2$ for difference (d.f. = 1) | | 4.05 ($P$=0.04) | | 3.97 ($P$=0.05) | | 0.08 ($P$=0.8) | | 4.04 ($P$=0.04) |
| Intra-uterine device | | | | | | | | |
| Never | 19 | 1.0 | 82 | 1.0 | 101 | 1.0 | 199 | 1.0 |
| Ever | 14 | 4.49 (1.66–13.0) | 39 | 0.86 (0.53–1.41) | 58 | 1.49 (0.99–2.23) | 111 | 1.38 (1.03–1.84) |
| $\chi^2$ for difference (d.f. = 1) | | 8.86 ($P$=0.003) | | 0.35 ($P$=0.6) | | 3.71 ($P$=0.05) | | 4.55 ($P$=0.03) |
| Diaphragm | | | | | | | | |
| Never | 31 | 1.0 | 106 | 1.0 | 130 | 1.0 | 264 | 1.0 |
| Ever | 2 | 0.34 (0.05–1.33) | 15 | 0.59 (0.32–1.09) | 29 | 0.64 (0.40–0.99) | 46 | 0.62 (0.43–0.88) |
| $\chi^2$ for difference (d.f. = 1) | | 2.28 ($P$=0.1) | | 3.13 ($P$=0.08) | | 3.95 ($P$=0.05) | | 7.80 ($P$=0.005) |
| Condom | | | | | | | | |
| Never | 20 | 1.0 | 71 | 1.0 | 102 | 1.0 | 193 | 1.0 |
| Ever | 13 | 0.83 (0.34–1.99) | 50 | 1.18 (0.77–1.81) | 57 | 0.96 (0.66–1.40) | 117 | 1.00 (0.76–1.30) |
| $\chi^2$ for difference (d.f. = 1) | | 0.18 ($P$=0.7) | | 0.59 ($P$=0.4) | | 0.05 ($P$=0.8) | | 0.001 ($P$=1.0) |
| Vasectomy | | | | | | | | |
| Never | 31 | 1.0 | 101 | 1.0 | 134 | 1.0 | 264 | 1.0 |
| Ever | 2 | 0.17 (0.02–0.70) | 20 | 0.71 (0.42–1.21) | 25 | 0.66 (0.41–1.05) | 46 | 0.62 (0.44–0.88) |
| $\chi^2$ for difference (d.f. = 1) | | 6.50 ($P$=0.01) | | 1.65 ($P$=0.2) | | 3.26 ($P$=0.07) | | 7.93 ($P$=0.005) |
| Tubal sterilisation | | | | | | | | |
| Never | 29 | 1.0 | 103 | 1.0 | 146 | 1.0 | 275 | 1.0 |
| Ever | 4 | 0.73 (0.16–2.46) | 18 | 1.13 (0.63–2.01) | 13 | 0.51 (0.27–0.94) | 35 | 0.73 (0.49–1.09) |
| $\chi^2$ for difference (d.f. = 1) | | 0.22 ($P$=0.6) | | 0.17 ($P$=0.7) | | 5.27 ($P$=0.02) | | 2.50 ($P$=0.1) |

[a]Number of cases in each category. For all variables all case–control sets contributed information to all the analyses. [b]Odds ratios and 95% confidence intervals adjusted for social class, smoking, age at first birth, ever use of OC, diaphragm and condom.

**Table III** Odds ratios (ORs) and 95% confidence intervals (CIs) of different categories of cervical neoplasia according to factors related to oral contraceptive use

| Variable | Invasive (33 case-control sets) Cases[a] | OR (95% CI)[b] | In situ (121 case-control sets) Cases[a] | OR (95% CI)[b] | Dysplasia (159 case-control sets) Cases[a] | OR (95% CI)[b] | All cervical neoplasia (311 case-control sets) Cases[a] | OR (95% CI)[b] |
|---|---|---|---|---|---|---|---|---|
| Total duration of OC use (months) | | | | | | | | |
| Never | 2 | 1.0 | 22 | 1.0 | 35 | 1.0 | 59 | 1.0 |
| 1–12 | 4 | | 4 | 1.41 (0.45–4.40) | 5 | 0.79 (0.30–2.14) | 13 | 1.37 (0.72–2.61) |
| 13–24 | 0 | 5.45 (0.79–50.5) | 7 | 1.75 (0.67–4.59) | 5 | 0.68 (0.24–1.89) | 12 | 1.09 (0.55–2.15) |
| 25–48 | 2 | | 20 | 1.71 (0.84–3.48) | 11 | 0.52 (0.25–1.10) | 32 | 0.96 (0.59–1.55) |
| 49–72 | 4 | 2.77 (0.49–23.1) | 18 | 1.48 (0.73–3.03) | 34 | 1.75 (1.02–3.02) | 54 | 1.66 (1.09–2.54) |
| 73–96 | 5 | | 11 | 1.15 (0.52–2.56) | 26 | 1.22 (0.68–2.19) | 42 | 1.31 (0.84–2.04) |
| 97+ | 16 | 4.65 (1.08–32.9) | 39 | 2.47 (1.30–4.68) | 43 | 1.06 (0.62–1.80) | 98 | 1.66 (1.13–2.45) |
| $\chi^2$ for trend (d.f. = 1)[c] | | 0.06 | | 1.35 | | 1.84 | | 3.15 |
| $P$-value | | 0.8 | | 0.2 | | 0.2 | | 0.08 |
| Months since last use of OCs | | | | | | | | |
| Never | 2 | 1.0 | 22 | 1.0 | 35 | 1.0 | 59 | 1.0 |
| Current–12 | 20 | | 45 | 2.22 (1.20–4.12) | 59 | 1.70 (1.02–2.83) | 124 | 2.23 (1.53–3.24) |
| 13–24 | 1 | 6.81 (1.56–49.2) | 2 | 0.56 (0.12–2.54) | 7 | 0.98 (0.40–2.40) | 10 | 0.76 (0.36–1.58) |
| 25–48 | 3 | | 12 | 1.81 (0.79–4.14) | 10 | 0.53 (0.25–1.14) | 24 | 0.92 (0.54–1.56) |
| 49–72 | 3 | 2.56 (0.43–21.3) | 12 | 1.95 (0.86–4.42) | 16 | 1.05 (0.54–2.04) | 29 | 1.25 (0.76–2.08) |
| 73–96 | 3 | | 9 | 1.45 (0.59–3.57) | 12 | 0.79 (0.38–1.65) | 24 | 1.09 (0.64–1.87) |
| 97+ | 1 | 1.30 (0.19–11.7) | 19 | 1.26 (0.59–2.70) | 20 | 0.71 (0.36–1.39) | 40 | 0.83 (0.51–1.34) |
| $\chi^2$ for trend (d.f. = 1)[c] | | 7.63 | | 1.69 | | 7.95 | | 17.2 |
| $P$-value | | 0.006 | | 0.2 | | 0.005 | | <0.0001 |

[a]Number of cases in each category. For all variables all case–control sets contributed information to all the analyses. [b]Adjusted for socal class, smoking, age at first birth, ever diaphragm use and ever condom use. [c]Trend in odds ratios across categories of ever users (see Materials and methods for details).

neoplasia and type of OC used by women at entry to the study and the most recent type of OC used in the 12 months before diagnosis. Of the 219 cases of cervical neoplasia who were OC users at entry, 209 were using OCs containing 50 μg oestrogen and seven were using high-oestrogen dose OC types (⩾100 μg oestrogen). The risk of all cervical neoplasia combined for 50 μg OCs (adjusted for age at first birth, smoking, social class and ever use of the diaphragm or condom) was similar to that for non-users at entry (OR = 1.07, 95% CI 0.78–1.47). Women using high-oestrogen combined OCs, however, appeared to have an elevated risk compared with non-users (OR = 2.62, 95% CI 0.99–6.96). For the 124 cases who had used OCs in the 12 months before diagnosis, two had last used one of unknown type, 25 had last used a progestogen only type, one had last used a combined type with ⩾100 μg oestrogen, 65 had last used combined OCs with 50 μg oestrogen and 31 had last used combined OCs with <50 μg oestrogen. The corresponding adjusted odds ratios for these groups (relative to never users) were 2.00 (95% CI 1.02–3.92) for progestogen only, 9.11 (95% CI 0.50–166.0) for combined OCs with ⩾100 μg oestrogen, 1.87 (95% CI 1.11–3.14) for 50 μg oestrogen and 1.53 (95% CI 0.85–2.73) for <50 μg oestrogen.

An important potential confounding variable which we could not take into account in the main analyses was frequency of cervical cytological smearing, as smear data were available only for the period of follow-up in the clinic. It was, however, possible to investigate at least in part the extent to which smear frequency might have affected the findings for OC use. Approximately 20% of both cases and controls were being followed up in the clinic at the date of diagnosis of the case and the median period of follow-up in the clinic was only slightly higher for cases than for controls (28 *vs* 24 months). Little difference was observed between cases and controls in median months of follow-up in the clinic until the first suspicious smear (if any was detected) (26 *vs* 24 months). Examination of smear frequency in 2647 control women with some clinic follow-up indicated no important differences between those using different methods of contraception at entry (not shown), ever/never use of OCs or different categories of time since last use of OCs (Table V). There was, however, a highly significant association between total duration of OC use and average interval between clinic smears ($\chi^2=123$, d.f.$=18$, $P<0.0001$) with a lower percentage of long-term users never having had a clinic smear (19.7% for 73–96 months and 14.9% for 97+ months compared with 25.8% for never users). However, after adjusting for time followed up in clinic until first suspicious smear (categorised in quartiles: 1–12, 13–26, 27–58 and 59+ months), no significant association was observed between duration of OC use and average clinic smear interval ($\chi^2=9.3$, d.f.$=18$, $P=0.95$). No differences in average smear interval were observed for other methods of contraception among controls with clinic follow-up (results not shown), apart from the fact that ever sterilised women had a lower smearing frequency than never sterilised women ($\chi^2=11.3$, d.f.$=3$, $P=0.01$).

### Discussion

In this nested case–control study of cervical cancer in women diagnosed before 45 years of age, ever users of OCs were found to be at an increased overall risk compared with never

**Table IV** Odds ratios for all cervical neoplasia combined (relative to never users) according to total duration of OC use and time since last OC use considered together

| | *Total duration of OC use (months)* | | | | | | | | | | | |
|---|---|---|---|---|---|---|---|---|---|---|---|---|
| | *1–24* | | *25–48* | | *49–72* | | *73–96* | | *97+* | | *Total* | |
| *Variable* | *Cases*[a] | *OR (95% CI)*[b] | *Cases*[a] | *OR (95% CI)* | *Cases*[a] | *OR (95% CI)*[b] | *Cases*[a] | *OR (95% CI)* | *Cases*[a] | *OR (95% CI)*[b] | *Cases*[a] | *OR (95% CI)*[b] |
| Time since last use (months) | | | | | | | | | | | | |
| Current–24 | 7 | 1.14 (0.46–2.79) | 10 | 0.91 (0.43–1.94) | 32 | 3.34 (1.96–5.67)* | 22 | 1.69 (0.97–2.95)† | 63 | 2.04 (1.34–3.11)‡ | 134 | 1.90 (1.32–2.74)** |
| 25–72 | 2 | 0.57 (0.13–2.42) | 9 | 1.46 (0.68–3.17) | 14 | 1.71 (0.90–3.26)† | 8 | 1.03 (0.47–2.29) | 20 | 0.92 (0.52–1.64) | 53 | 1.10 (0.72–1.69) |
| 73+ | 16 | 1.42 (0.75–2.68) | 13 | 0.81 (0.42–1.58) | 8 | 0.52 (0.24–1.15) | 12 | 1.00 (0.50–2.03) | 15 | 1.23 (0.63–2.41) | 64 | 0.94 (0.61–1.45) |
| Total | 25 | 1.22 (0.73–2.04) | 32 | 0.96 (0.59–1.55) | 54 | 1.67 (1.09–2.55)† | 42 | 1.31 (0.84–2.05) | 98 | 1.66 (1.13–2.46)‡‡ | 251 | 1.40 (1.00–1.96)*** |

[a]Number of cases in each subcategory. [b]Adjusted for social class, age at first birth, smoking, ever use of diaphragm and ever use of condom. *$\chi^2=19.9$ ($P<0.001$), †$\chi^2=3.4$ ($P=0.07$), ‡$\chi^2=11.0$ ($P<0.001$), **$\chi^2=12.0$ ($P<0.001$), ††$\chi^2=5.61$ ($P=0.02$), ‡‡$\chi^2=6.62$ ($P=0.01$), ***$\chi^2=4.04$ ($P=0.04$). All other $P$-values were ⩾0.1.

**Table V** Number of controls with a clinic follow-up ($n=2647$) according to average interval between smears and method of contraception (row percentage in parentheses)

| | | *Average smear interval*[a] | | | | |
|---|---|---|---|---|---|---|
| *Variable* | | *⩽1 year* | *2–3 years* | *4+ years* | *Never smear* | *Total (100%)* |
| Total number | | 1005 | 811 | 158 | 673 | 2647 |
| OC use* | | | | | | |
| | Never | 257 (38.5%) | 198 (29.6%) | 41 (6.1%) | 172 (25.8%) | 668 |
| | Ever | 748 (37.8%) | 613 (31.0%) | 117 (5.9%) | 501 (25.3%) | 1979 |
| Total duration of OC use (months)† | | | | | | |
| | Never | 257 (38.5%) | 198 (29.6%) | 41 (6.1%) | 172 (25.8%) | 668 |
| | 1–12 | 29 (31.2%) | 22 (23.7%) | 6 (6.5%) | 36 (38.7%) | 93 |
| | 13–24 | 41 (35.0%) | 20 (17.1%) | 1 (0.9%) | 55 (47.0%) | 117 |
| | 25–48 | 147 (37.5%) | 93 (23.7%) | 15 (3.8%) | 137 (35.0%) | 392 |
| | 49–72 | 151 (38.9%) | 110 (28.4%) | 19 (4.9%) | 108 (27.8%) | 388 |
| | 73–96 | 139 (37.6%) | 136 (36.8%) | 22 (6.0%) | 73 (19.7%) | 370 |
| | 97+ | 241 (38.9%) | 232 (37.5%) | 54 (8.7%) | 92 (14.9%) | 619 |
| Time since last OC use (months)‡ | | | | | | |
| | Never | 257 (38.5%) | 198 (29.6%) | 41 (6.1%) | 172 (25.8%) | 668 |
| | Current–12 | 276 (39.3%) | 216 (30.8%) | 35 (5.0%) | 175 (24.9%) | 702 |
| | 13–24 | 49 (36.3%) | 43 (31.9%) | 10 (7.4%) | 33 (24.4%) | 135 |
| | 25–48 | 99 (36.4%) | 92 (33.8%) | 21 (7.7%) | 60 (22.1%) | 272 |
| | 49–72 | 95 (39.3%) | 71 (29.3%) | 13 (5.4%) | 63 (26.0%) | 242 |
| | 73–96 | 75 (35.9%) | 67 (32.1%) | 15 (7.2%) | 52 (24.9%) | 209 |
| | 97+ | 154 (36.8%) | 124 (29.6%) | 23 (5.5%) | 118 (28.2%) | 419 |

[a]Computed as follow-up period in clinic before any suspicious smear divided by number of normal smears in that period. Controls without any clinic follow-up were excluded ($n=444$). [b]Number of controls (row percentage). *$\chi^2=0.43$, d.f.$=3$, $P=0.9$; †$\chi^2=123$, d.f.$=18$, $P<0.0001$; ‡$\chi^2=9.58$, d.f.$=18$, $P=0.9$.

users, with excess risks appearing to be highest for invasive cancer, intermediate for carcinoma *in situ* and lowest for dysplasia. Ever use of the diaphragm, female tubal sterilisation and vasectomy of the husband were associated with a reduced overall risk of cervical neoplasia, whereas ever use of IUDs was associated with a slightly increased overall risk of the disease. For OC use, the increased risk was largely confined to recent, long-term users. Long-term use in the past (when OCs had been stopped more than 24 months before date of diagnosis) did not seem to be associated with an increased risk. All risk estimates were adjusted for variables that were defined a priori as having important potential confounding effects: smoking, social class, age at first birth and ever use of diaphragm or condom. Additional adjustments for ever use of other contraceptive methods and for ever having undergone an induced abortion during follow-up tended to strengthen the significance of the results for OCs.

Long-term and/or recent OC use have been associated separately with an increased risk of invasive cervical carcinoma and carcinoma *in situ* in the Royal College of General Practitioners' cohort study (Beral *et al.*, 1988). Similar findings have been reported from several large case–control studies that examined the relationship between OCs and invasive cancer (WHO, 1985, 1993; Brinton *et al.*, 1986, 1987; Parazzini *et al.*, 1990) or carcinoma *in situ* (Irwin *et al.*, 1988; Jones *et al.*, 1990; Kjaer *et al.*, 1993; Ye *et al.*, 1995). Some smaller studies have reported no significant findings for long-term or recent OC use in relation to invasive cancer (Peters *et al.*, 1986; Ebeling *et al.*, 1987; Irwin *et al.*, 1988; Cuzick *et al.*, 1989) or carcinoma *in situ* (Molina *et al.*, 1988; Becker *et al.*, 1994), and it has been suggested that the previously reported results relating OC use to cervical neoplasia were caused by detection bias and lack of control for confounding (Irwin *et al.*, 1988). Two large case–control studies investigated the combined effect of duration and recency of OC use on risk of invasive carcinoma of the cervix (Brinton *et al.*, 1990; WHO 1993). In agreement with our results, these studies also reported the highest relative risks in current/recent, long-term users of OCs. Results concerning the association between OC use and the risk of dysplasia have been inconclusive (Negrini *et al.*, 1990; De Vet *et al.*, 1993). Our results suggest that OC use may not be as strongly associated with risk of dysplasia as with risk of invasive carcinoma or carcinoma *in situ*.

In this study we also observed a reduced risk of cervical neoplasia associated with use of the diaphragm, female tubal sterilisation and vasectomy of the husband. Use of barrier methods has been reported consistently as a protective factor for cervical cancer in previous studies (Parazzini *et al.*, 1989; Coker *et al.*, 1992). Unexpectedly, we found no effect of use of the condom. A likely explanation for this is that condom users relied on this contraceptive method for relatively short periods of time compared with diaphragm users, since none of the women relying on condoms were using this method at entry to the study. A protective effect of female tubal sterilisation and of vasectomy has also been reported previously (Swan and Brown, 1979, 1981). The possible effect of the latter method has been interpreted as being due to the removal of sperm basic proteins that have been suggested as playing a role in cervical carcinogenesis (Reid *et al.*, 1978).

The significantly increased risk of cervical neoplasia for ever users of IUDs that was most evident for invasive carcinoma (Table II), appeared to be largely explained by the influence of other contraceptive methods and the effect of induced abortion during follow-up. Most other studies that have investigated IUD use have found no significant effect on cervical neoplasia (Peters *et al.*, 1986; Cuzick *et al.*, 1989; Lassise *et al.*, 1991), although a protective effect has been reported in two (Molina *et al.*, 1988; Brinton *et al.*, 1990).

In our previous cohort analysis we reported that nine out of 13 cases of invasive cervical cancer were found among long-term OC users (more than 72 months of use), that both carcinoma *in situ* and dysplasia also occurred more frequently in the oral contraceptive entry group than in the IUD entry group, and that there was a significant trend in incidence of all three forms of cervical neoplasia combined with increasing duration of OC use (Vessey *et al.*, 1983). The present nested case–control study has several advantages over the previous cohort analysis and over case–control studies that are not nested within a cohort. Compared with our previous analysis, there has been a large increase in the number of cases available, which has made the present analyses more stable and the findings less likely to be due to chance. The case–control approach enabled us to consider OC use in much more detail than before, while adjusting for a number of important potential confounding variables. Furthermore, unlike non-nested case–control studies, the data in contraceptive use and confounding variables were collected during annual follow-up and were therefore much less prone to recall bias.

One limitation of our study was that we were unable to adjust for the potentially important confounding effects of number of sexual partners and age at first intercourse. We did adjust for the effect of age at first birth (which was strongly related to cervical neoplasia risk) which, in this cohort of women married for the most part in the 1960s or earlier, may have been closely related to age at first intercourse. Age at marriage, which might also be expected to be related to factors such as age at first intercourse and number of sexual partners, was not related to cervical cancer risk in this study after adjustment for age at first birth (Table I). Data collected in 1977 for a small subset of women in the cohort (58 with cervical neoplasia and 139 matched controls) found that women who were IUD users or OC users at entry did not differ with respect to age at first intercourse or number of sexual partners, whereas diaphragm users at entry were less likely to have had intercourse at an early age and had had fewer sexual partners (Wright *et al.*, 1978). All women who entered the study were married, and the majority (69%) reported having had only one sexual partner. Strong confounding by number of sexual partners or age at first intercourse therefore seems unlikely in this study, although we cannot rule out the possibility that some residual confounding owing to these factors may have influenced the results.

Recently, infection with some subtypes of the human papillomavirus (16 and 18 in particular) has been strongly associated with risk of invasive cervical cancer in two case–control studies (Bosch *et al.*, 1992; Eluf-Neto *et al.*, 1994). These studies, however, were conducted in settings where the prevalence of HPV infection of women was relatively high. Owing to very small numbers of HPV-negative cases and HPV-positive controls the independent effect of OC use on risk of cervical cancer could not be accurately determined. Risk of HPV infection is strongly associated with early age at first intercourse and in particular with the number of sexual partners (Eluf-Neto *et al.*, 1994). As mentioned earlier, the majority of the women in our study were likely to have had only one sexual partner and therefore the risk of HPV infection in these women may be relatively low. It should be noted, however, that we have no data at all on number of sexual partners of the husband that may have influenced the risk of HPV infection for the women in this study.

It was not possible to adjust our results for frequency of cervical cytological smearing since these data were available only during clinic follow-up. We were able, however, to investigate the potential confounding effect of this variable to some extent by examining the association between the average smear interval during clinic follow-up and use of oral and other methods of contraception using data for controls. Long-term users of OCs (over 72 months) had at least one clinic smear significantly more frequently than never or short-term users, but after adjusting for the period of follow-up in the clinic (which was relatively longer for long-term users of OCs) the statistical significance of this association disappeared. No differences were found across categories of time since last use of OCs, ever/never use of OCs and ever/never use of different methods of contraception at entry to the cohort. Although we cannot be sure that clinic smear frequency for controls was representative of the smear frequency during the total follow-up period, these data provide some reassurance regarding the chance of a detection bias having occurred. Moreover, if a detection bias had been operating, and OC users had undergone cervical smearing more frequently than users of other methods, this would have tended to increase the chances of diagnosis of less severe lesions (Hulka, 1989). The highest risk associated with OC use was observed in the invasive carcinoma group, whereas the lowest risk was observed in the dysplasia group. This pattern is not likely to be explained by a detection bias.

The results of this nested case–control study largely confirm the findings of our previous report (Vessey *et al.*, 1983), but provide a better insight into the relationship between different aspects of OC use and cervical neoplasia. The results suggest that current/recent, long-term users of OCs may have an elevated risk of cervical neoplasia, in particular of invasive carcinoma and carcinoma *in situ*. Although we cannot rule out residual confounding by sexual factors and we could not adjust for the possible effect of HPV infection, these findings provide independent support for an effect of OCs which appears to operate in the later stages of cervical carcinogenesis.

### References

BECKER TM, WHEELER CM, MCGOUGH NS, STIDLEY CA, PARMENTER CA, DORIN MH AND JORDAN SW. (1994). Contraceptive and reproductive risks for cervical dysplasia in southwestern Hispanic and non-Hispanic white women. *Int. J. Epidemiol.*, **23,** 913–922.

BERAL V, HANNAFORD P AND KAY C. (1988). Oral contraceptive use and malignancies of the genital tract. Results from the Royal College of General Practitioners' Oral Contraception Study. *Lancet*, **121,** 1331–1335.

BOSCH FX, MUNOZ N, DE SANJOSÉ S, IZARZUGAZA I, GILI M, VILADIU P, TORMO MJ, MOREO P, ASCUNCE N, GONZALEZ LC, TAFUR L, KALDOR JM, GUERRERO E, ARISTIZABAL N, SANTAMOVIA M AND ALFONSODE RUIZ P. (1992). Risk factors for cervical cancer in Colombia and Spain. *Int. J. Cancer*, **52,** 750–758.

BRESLOW NE AND DAY NE. (1980). *Statistical Methods in Cancer Research*, **Vol. 1,** *The Analysis of Case–Control Studies.* IARC Scientific Publications No. 32, International Agency for Research on Cancer: Lyon.

BRINTON LA, HUGGINS GR, LEHMAN HF, MALLIN K, SAVITZ DA, TRAPIDO E, ROSENTHAL J AND HOOVER R. (1986). Long-term use of oral contraceptives and risk of invasive cervical cancer. *Int. J. Cancer*, **38,** 339–344.

BRINTON LA, TASHIMA KT, LEHMAN HF, LEVINE RS, MALLIN K, SAVITZ DA, STOLLEY PD AND FRAUMENI JF Jr. (1987). Epidemiology of cervical cancer by cell type. *Cancer Res.*, **47,** 1706–1711.

BRINTON LA, REEVES WC, BRENES MM, HERRERO R, DE BRITTON RC, GAITAN E, TENORIO F, GARCIA M AND RAWLS WE. (1990). Oral contraceptive use and risk of invasive cervical cancer. *Int. J. Epidemiol.*, **19,** 4–11.

COKER AL, HULKA BS, MCCANN MF AND WALTON LA. (1992). Barrier methods of contraception and cervical intraepithelial neoplasia. *Contraception*, **45,** 1–10.

CUZICK J, DE STAVOLA B, MCCANCE D, HO TH, TAN G, CHENG H, CHEW SY AND SALMON YM. (1989). A case–control study of cervix cancer in Singapore. *Br. J. Cancer*, **60,** 238–243.

DE VET HCW, KNIPSCHILD PG AND STURMANS F. (1993). The role of sexual factors in the aetiology of cervical dysplasia. *Int. J. Epidemiol.*, **22,** 798–803.

EBELING K, NISCHAN P AND SCHINDLER C. (1987). Use of oral contraceptives and risk of invasive cervical cancer in previously screened women. *Int. J. Cancer*, **39,** 427–430.

ELUF-NETO J, BOOTH M, MUNOZ N, BOSCH FX, MEIJER CJLM AND WALBOOMERS JMM. (1994). Human papillomavirus and invasive cervical cancer in Brazil. *Br. J. Cancer*, **69,** 114–119.

GREENBERG ER, VESSEY M, MCPHERSON K AND YEATS D. (1985). Cigarette smoking and cancer of the uterine cervix. *Br. J. Cancer*, **51,** 139–141.

HIROSOFT INTERNATIONAL CORPORATION. (1990). *EPICURE.* Seattle, WA, USA.

HULKA BS. (1989). Hormonal contraceptives and risk of cervical cancer. In *Safety Requirements for Contraceptive Steroids*, Michal F (ed.) pp. 84–96. WHO/Cambridge University Press: Cambridge.

IRWIN KL, ROSERO-BIXBY L, OBERLE MW, LEE NC, WHATLEY AS, FORTNEY JA AND BONHOMME MG. (1988). Oral contraceptives and cervical cancer risk in Costa Rica. Detection bias or causal association? *JAMA*, **259,** 59–64.

JONES CJ, BRINTON LA, HAMMAN RF, STOLLEY PD, LEHMAN HF, LEVINE RS AND MALLIN K. (1990). Risk factors for *in situ* cervical cancer: results from a case–control study. *Cancer Res.*, **50,** 3657–3662.

KJAER SK, ENGHOLM G, DAHL C, BOCK JE, LYNGE E AND JENSEN OM. (1993). Case–control study of risk factors for cervical squamous-cell neoplasia in Denmark III. Role of oral contraceptive use. *Cancer Causes Control*, **4,** 513–519.

LASSISE DL, SAVITZ DA, HAMMAN RF, BARON AE, BRINTON LA AND LEVINES RS. (1991). Invasive cervical cancer and intrauterine device use. *Int. J. Epidemiol.*, **20,** 865–870.

MOLINA R, THOMAS DB, DABANCENS A, LOPEZ J, RAY RM, MARTINEZ L AND SALAS O. (1988). Oral contraceptives and cervical carcinoma *in situ* in Chile. *Cancer Res.*, **48,** 1011–1015.

NEGRINI BP, SCHIFFMAN MH, KURMAN RJ, BARNES W, LANNOM L, MALLEY K, BRINTON LA, DELGADO G, JONES S AND TCHABO, JG. (1990). Oral contraceptive use, human papillomavirus infection, and risk of early cytological abnormalities of the cervix. *Cancer Res.*, **50,** 4670–4675.

PARAZZINI F, NEGRI E, LA VECCHIA C AND FEDELE L. (1989). Barrier methods of contraception and the risk of cervical neoplasia. *Contraception*, **40,** 519–530.

PARAZZINI F, LA VECCHIA C, NEGRI E AND MAGGI R. (1990). Oral contraceptive use and invasive cervical cancer. *Int. J. Epidemiol.*, **19,** 259–263.

PETERS RK, THOMAS D, HAGAN DG, MACK TM AND HENDERSON BE. (1986). Risk factors for invasive cervical cancer among Latinas and non-Latinas in Los Angeles County. *J. Natl Cancer Inst.*, **77,** 1063–1077.

REID BL, FRENCH PW, SINGER A, HAGAN BE AND COPPLESON M. (1978). Sperm basic proteins in cervical carcinogenesis: correlation with socioeconomic class. *Lancet*, **2,** 60–62.

STATISTICS AND EPIDEMIOLOGICAL RESEARCH CORPORATION. (1989). *EGRET User Manual.* Seattle, WA, USA.

SWAN SH AND BROWN WL. (1979). Vasectomy and cancer of the cervix. *N. Engl. J. Med.*, **301,** 46.

SWAN SH AND BROWN WL. (1981). Oral contraceptive use, sexual activity, and cervical carcinoma. *Am. J. Obstet. Gynecol.*, **139,** 52–57.

SWAN SH AND PETITTI DB. (1982). A review of problems of bias and confounding in epidemiologic studies of cervical neoplasia and oral contraceptive use. *Am. J. Epidemiol.*, **115,** 10–18.

VESSEY M, DOLL R, PETO R, JOHNSON B AND WIGGINS P. (1976). A long-term follow up study using different methods of contraception–an interim report. *J. Biosoc. Sci.*, **8,** 373–427.

VESSEY MP, LAWLESS M, MCPHERSON K AND YEATES D. (1983). Neoplasia of the cervix uteri and contraception: a possible adverse effect of the pill. *Lancet*, **2,** 930–934.

WHO COLLABORATIVE STUDY OF NEOPLASIA AND STEROID CONTRACEPTIVES. (1985). Invasive cancer and combined oral contraceptives. *Br. Med. J.*, **290,** 961–965.

WHO COLLABORATIVE STUDY OF NEOPLASIA AND STEROID CONTRACEPTIVES. (1993). Invasive squamous-cell cervical carcinoma and combined oral contraceptives: results from a multi-national study. *Int. J. Cancer*, **55,** 228–236.

WRIGHT NH, VESSEY MP, KENWARD B, MCPHERSON K AND DOLL R. (1978). Neoplasia and dysplasia of the cervix uteri and contraception, a possible protective effect of the diaphragm. *Br. J. Cancer*, **38,** 273–279.

YE Z, BAY TDB, RAY RM AND THE WHO COLLABORATIVE STUDY OF NEOPLASIA AND STEROID CONTRACEPTIVES. (1995). Combined oral contraceptives and risk of cervical carcinoma *in situ*. *Int. J. Epidemiol.*, **24,** 19–26.